# Old age promotes retinal fibrosis in choroidal neovascularization through circulating fibrocytes and profibrotic macrophages

**DOI:** 10.1186/s12974-023-02731-y

**Published:** 2023-02-23

**Authors:** Caijiao Yi, Jian Liu, Wen Deng, Chang Luo, Jinyan Qi, Mei Chen, Heping Xu

**Affiliations:** 1 Aier School of Ophthalmology, Central South University, Changsha, 410000 China; 2 Aier Institute of Optometry and Vision Science, Changsha, 410000 China; 3 Department of Ophthalmology, The Second Xiangya Hospital, Central South University, Changsha, 410011 China; 4 Hunan Province Optometry Engineering and Technology Research Center, Changsha, 410009 China; 5 Hunan Province International Cooperation Base for Optometry Science and Technology, Changsha, 410009 China; 6 The Wellcome-Wolfson Institute for Experimental Medicine, School of Medicine, Dentistry and Biomedical Sciences, Queen’s University Belfast, 97 Lisburn Road, Belfast, BT9 7BL UK

**Keywords:** Age-related macular degeneration, Bone marrow, Inflammation, Macular fibrosis

## Abstract

**Background:**

Retinal fibrosis affects 40–70% of neovascular age-related macular degeneration patients. This study investigated the effect of ageing on subretinal fibrosis secondary to choroidal neovascularization and the mechanism of action.

**Methods:**

Subretinal fibrosis was induced in young (2.5-month) and aged (15–16-month) C57BL/6J mice using the two-stage laser protocol. Five and 30 days later, eyes were collected and stained for CD45 and collagen-1 and observed by confocal microscopy. Fibrocytes (CD45^+^collagen-1^+^) were detected in the bone marrow (BM), blood and fibrotic lesions by flow cytometry and confocal microscopy, respectively. BM-derived macrophages (BMDMs) were cultured from young and aged mice with or without TGF-β1 (10 ng/mL) treatment. The expression of mesenchymal marker αSMA (*Acta2*), fibronectin (*Fn1*) and collagen-1 (*Col1a1*) was examined by qPCR and immunocytochemistry, whereas cytokine/chemokine production was measured using the Luminex multiplex cytokine assay. BM were transplanted from 22-month (Ly5.2) aged mice into 2.5-month (Ly5.1) young mice and vice versa. Six weeks later, subretinal fibrosis was induced in recipient mice and eyes were collected for evaluation of fibrotic lesion size.

**Results:**

Under normal conditions, the number of circulating fibrocytes (CD45^+^collagen-1^+^) and the expression levels of *Tgfb1, Col1a1, Acta2* and *Fn1* in BMDMs were significantly higher in aged mice compared to young mice. Induction of subretinal fibrosis significantly increased the number of circulating fibrocytes, enhanced the expression of *Col1a1, Acta2* and *Fn1* and the production of soluble urokinase plasminogen activator surface receptor (suPAR) but decreased the production of CXCL10 in BMDMs. BMDMs from aged subretinal fibrosis mice produced significantly higher levels of VEGF, angiopoietin-2 and osteopontin than cells from young subretinal fibrosis mice. The subretinal fibrotic lesion in 15–16-month aged mice was 62% larger than that in 2.5-month young mice. The lesion in aged mice contained a significantly higher number of fibrocytes compared to that in young mice. The number of circulating fibrocytes positively correlated with the size of subretinal fibrotic lesion. Transplantation of BM from aged mice significantly increased subretinal fibrosis in young mice.

**Conclusions:**

A retina–BM–blood–retina pathway of fibrocyte/macrophage recruitment exists during retinal injury. Ageing promotes subretinal fibrosis through higher numbers of circulating fibrocytes and profibrotic potential of BM-derived macrophages.

**Supplementary Information:**

The online version contains supplementary material available at 10.1186/s12974-023-02731-y.

## Introduction

Fibrosis is the excessive scar formation resulting from abnormal wound healing and is characterized by extensive extracellular matrix deposition, chronic inflammation and progressive tissue/organ remodeling. Fibrosis disrupts the normal tissue architecture and impairs organ function. Fibrosis-mediated organ failure (e.g., lung, kidney, liver and heart) is the leading cause of morbidity and mortality accounting for ~ 45% of all causes of death in the United States [1]. Retinal fibrosis can develop in the conditions, such as proliferative diabetic retinopathy, rhegmatogenous retinal detachment, neovascular age-related macular degeneration (nAMD), and choroidal neovascularization (CNV) secondary to other eye diseases (e.g., myopic retinopathy or choroiditis). Retinal fibrosis is the leading cause of irreversible vision loss [2]. Despite the tremendous clinical impact of fibrosis, effective therapies are extremely limited, largely due to a poor understanding of disease mechanisms [3].

Ageing is one of the major risk factors for fibrosis. The incidence of organ fibrosis, such as pulmonary fibrosis [4], liver cirrhosis [5], renal fibrosis [6], and cardiac fibrosis [7] increases with age. Macular fibrosis is seen predominately in nAMD, which affects people older than 55 years of age [8]. nAMD is treated with intraocular injection of vascular endothelial growth factor (VEGF) inhibitors (e.g., aflibercept, ranibizumab, and conbercept) [9]. Before the use of VEGF inhibitor therapy, almost all nAMD eventually progressed to macular fibrosis [8]. Nowadays, with anti-VEGF therapy, between 40 and 70% of nAMD patients develop macular subretinal fibrosis within 10 years[10–12].

In addition to nAMD, fibrovascular membrane can also develop in other ocular diseases, such as retinopathy of prematurity, proliferative vitreoretinopathy or diabetic retinopathy, uveitis, and pathological myopia [13–15]. The risk of CNV progressing into fibrous lesion increases with age. Approximately 40% of myopic CNV eyes may develop subretinal fibrosis after anti-VEGF therapy [15], but patients younger than 40 years of age usually respond well to anti-VEGF therapy and are less likely to develop subretinal fibrosis [16].

It is unclear why ageing increases the risk of organ fibrosis. Classic wound healing starts with inflammation, followed by proliferation and remodeling. The process is regulated by both the microenvironment of the affected tissue/organ and systemic factors, particularly the immune system. Ageing is accompanied by a progressive decline of certain immune functions and low-grade inflammation (inflammaging) [17]. Aged people are more susceptible to infection and are at a greater risk of developing inflammatory diseases as evidenced by the fact that the elderly are affected more badly than other age groups during the current COVID-19 pandemic [18]. During wound healing, immune cells, in particular macrophages, are important scavengers to clean up the wound by removing damaged or dead cells and debris [19]. Macrophages along with fibrocytes also play a critical role in the proliferation and remodeling stages of wound healing [19, 20]. Ageing is known to affect many functions of macrophages including phagocytosis, cytokine production, antigen presentation, migration etc. [21], although how this is linked to organ fibrosis is less known. Fibrocytes are hematopoietic-derived collagen-1-producing cells. Once formed in the bone marrow (BM), they move to blood circulation and can migrate to the spleen and peripheral tissues [22]. When an injury occurs, fibrocytes are recruited and differentiate into myofibroblasts, which contribute to wound healing and fibrosis [22]. Although there is no consensus on fibrocytes-specific markers, the cells are generally identified by their hematopoietic origin (e.g., express CD34 and CD45) and their ability to produce collagen-1/3 [22]. Altered circulating fibrocytes have been linked to idiopathic pulmonary fibrosis and atrial fibrosis [23–25], although their function is affected by age and their role in age-related retinal fibrosis remain unknown.

We previously developed a two-stage laser-induced subretinal fibrosis protocol in mice [26]. This model mimics closely the pathology of macular fibrosis secondary to CNV [26] and is an ideal tool to study the pathogenesis of subretinal fibrosis. Here, using this model, we investigated how ageing affects retinal wound healing and subretinal fibrosis and the mechanisms of action with a particular focus on age-related alterations in bone-marrow-derived cells, including circulating fibrocytes and macrophages.

## Materials and methods

### Animals

C57BL/6J-Ly5.2 (CD45.2) mice (2.5 months) were purchased from the SJA Laboratory Animal Co., Ltd (Changsha, China). C57BL/6J-Ly5.1 (CD45.1) mice (2.5 months) were provided by Junke Biological Co., Ltd (Nanjing, China). All animals were maintained in specific pathogen-free (SPF) conditions on a 12-h day/night cycle with free access to food and water in the Department of Laboratory Animals of Hunan Normal University, China. Some C57BL/6J-Ly5.2 (CD45.2) mice were maintained to 15–16 months before experimentation. All animal-related procedures were conducted following the Association for Research in Vision and Ophthalmology (ARVO) Statement for the Use of Animals in Ophthalmic and Vision Research and the protocols were approved by the Animal Welfare Ethics Committee of Hunan Normal University (Ref no: D2019017).

### Whole-body irradiation and bone-marrow transplantation (BMT)

We conducted two groups of BMT, “young (2.5-month-old C57BL/6J-Ly5.1 (CD45.1)) → old (22-month-old C57BL/6J-Ly5.2 (CD45.2))” and “old → young”. The recipient mice were fed with sterilized food and water containing antibiotic gentamicin sulfate (Yiduoli Co., Ltd, Shanxi, China) 1 week before whole-body irradiation (12 Gy, 2 × 6 Gy, 20 h apart) using Precision X-Ray 225 Irradiator (Connecticut, USA). In the “old → young” group, 1 × 10^7^ bone-marrow cells from 22-month-old C57BL/6J-Ly5.2 (CD45.2) donor mice were transplanted into the young Ly5.1 (CD45.1) irradiated recipient mice via tail vein injection (150 µl/mouse) within 4 h after irradiation. The same number of cells from young Ly5.1 (CD45.1) mice were transplanted into irradiated old C57BL/6J-Ly5.2 (CD45.2) mice in the “young → old” group. Mice were fed with sterilized food and antibiotic water for 3 weeks. Body weights were monitored every other day. Assessment of immune system reconstitution was conducted in 20 µl blood 4 and 6 weeks later by flow cytometry. The mice were then subjected to subretinal fibrosis induction.

### Induction of subretinal fibrosis

A two-stage laser protocol detailed previously by us [26, 27] was used to induce subretinal fibrosis in young (2.5–3 months or 4 months in BMT study) and aged (15–16 months and 24 months in BMT) mice. Briefly, mice were anaesthetized with an intraperitoneal injection of sodium pentobarbital (60 mg/kg, Sigma Aldrich, St. Louis, MO), and pupils were dilated with 0.5% tropicamide and 0.5% phenylephrine (Santen Pharmaceutical Co., Ltd., Osaka, Japan). Each eye received four laser burns (Topcon, Tokyo, Japan, 200 mv power, 100 ms duration and 60 μm spot size). Ten days later, a second laser burn was applied to each lesion using the same setting. Eyes were collected 5 and 30 days after the second laser and processed for further analysis.

### Flow cytometry

#### Fibrocyte detection

Bone marrow cells (3 × 10^6^) and blood samples (50 µl) were incubated with mouse Fc receptor block solution (Cat: 553141, BD Biosciences, San Diego, CA, USA) for 10 min at 4 °C. The samples were then incubated with brilliant violet 421 (BV421)-conjugated rat anti-mouse CD45 (1:100, Cat: 563890, BD Biosciences,) or isotype control BV421-Rat IgG2bκ (1:100, Cat: 562603, BD Biosciences) for 30 min in the dark at 4 °C. After washing with PBS, the samples were incubated with lysis buffer (Cat: 00-4300-54, Invitrogen, MA, USA) for 10 min. Samples were then washed, fixed and permeabilized with pre-cold methanol on ice for 10 min followed by incubation with 10% goat serum for 20 min. The cells were incubated with rabbit anti-collagen-1 (1:400, Cat: ab34710, Abcam, Cambridge, UK) or isotype control rabbit IgG (1:400, Cat: ab171870, Abcam), followed by incubation with Alexa Fluor 488 goat anti-rabbit IgG (1:800, Cat. No: A11034, Invitrogen) for 30 min.

#### BMT confirmation

Mice tail blood (20 µl) was harvested 4 and 6 weeks after BMT. The blood samples were incubated with Allophycocyanin (APC)-conjugated rat anti-mouse CD45.1 (1:100, Cat:110714, Biolegend, CA, USA) and Phycoerythrin (PE)-conjugated anti-mouse CD45.2 (1:100, Cat: 109807, Biolegend) for 30 min in the dark at 4 °C, followed by red blood cell removal with lysis buffer.

All samples were acquired on the FACSCelesta flow cytometer (BD Biosciences) and data were analysed using the FlowJo software (Version 10, Tree Star, Oregon, USA). The gating strategies were based on relevant isotype controls. CD45^+^Collagen-1^+^ cells were considered fibrocytes.

### Isolation and culture of bone-marrow cells

BM cells from young and aged mice with/without subretinal fibrosis were isolated and cultured as previously described [28, 29]. In brief, after flushing BM cells from the tibias and femurs, the red blood cells were removed with lysis buffer, and the cells were cultured in DMEM (Cat: 8121516, Gibco, Grand Island, NY, USA) supplemented with 20% L929 supernatant, 15% fetal bovine serum (FBS, Cat: 10099141C, Gibco), and 100 mg/ml primocin (Cat: PML-41-06, Invivogen, CA, USA) for 5–7 days to generate bone-marrow derived-macrophages (BMDMs). Flow cytometry confirmed that > 95% of BMDM were CD11b^+^F4/80^+^.

BMDMs from each group were treated with 10 ng/ml recombinant mouse TGF-β1 for 96 h (Cat: 7666-MB-005, R&D Systems, Minneapolis, MN, USA). The cells were then thoroughly washed and cultured in serum-free DMEM for another 24 h. Supernatants were used for Luminex multiplex cytokine assay. Cells were collected for immunocytochemistry or RT-PCR.

### Quantitative real-time PCR (qRT-PCR)

Total RNA was extracted from BMDMs using the total RNA Kit II (Cat: R6934-01, Omega, Norcross, GA) according to the manufacturer’s instructions. cDNA was synthesized using the PrimeScript RT Reagent Kit (Cat: 6110A, Vazyme Biotech, Nanjing, China). Quantitative real-time PCR was performed in a total of 10 µl mixture solution using the LightCycler 96 (Roche, Basel, Switzerland). Each 10 µl reaction mixture contains 5 µl SYBR Green PCR MasterMix (Cat: Q711-02, Vazyme Biotech), 1 µM primers and diluted cDNA. *GAPDH* was used as a housing-keeping gene. The primers referenced in this study were designed using the NCBI Primer BLAST system and purchased from TSINGKE (Changsha, Hunan, China). The primer sequences are detailed in Additional file 1: Table S1.

### Luminex multiplex cytokine assay

Luminex multiplex beads assay (Cat: LXSAMSM-15, R&D Systems) was conducted to measure the production of 15 cytokines (CCL2, VEGF, CXCL10, Osteopontin (OPN), Angiopoietin-2 (Ang-2), uPAR, PDGFα, PDGFβ, IL-1α, IL-1β, IL-10, CD105, EGF, GM-CSF, G-CSF) in BMDM supernatants from different groups according to the manufacturer’s instructions. In brief, supernatants were incubated with colour-coded beads pre-coated with 15 capture antibodies for 2 h at room temperature. 50 µl of biotinylated detection antibodies specific to the analytes of interest were added and incubated for 1 h, followed by incubation with PE-conjugated streptavidin for 30 min. Samples were measured using the Luminex 200™ analyzer (Luminex, Austin, TX, USA). The levels of cytokines and chemokines were normalized by the total protein of the supernatants (determined by BCA assay, Cat: PC0020, Solarbio, Beijing, China).

### Immunofluorescence

#### Mouse eye section staining

Mouse eyes were enucleated at 5 and 30 days post the second laser and fixed in 4% paraformaldehyde (PFA) for 4 h at room temperature. The eyes were embedded in optimal cutting temperature (OCT) and cryosections were prepared. The sections were blocked with 10% goat serum in 2% BSA and permeabilized with 0.1% Triton X-100 for 1 h at room temperature, followed by incubation with primary antibodies. The primary antibodies used in this study include rat anti-mouse CD45 (1:100, Cat: 14045182, Invitrogen), rat anti-mouse F4/80 ((1:200, Cat: ab6640, Abcam) and rabbit anti-mouse collagen-1 (1:200, Abcam), rat anti-mouse CD31 (1:100, Cat: MA1-40074, Invitrogen) and rabbit anti-mouse collagen-III (1:200, Cat: 22734-1-AP, Proteintech) at 4 °C overnight. After thorough washes, samples were incubated with Alexa Fluor 488-conjugated donkey anti-rat IgG (1:200, Cat: A48269, Invitrogen) and Alexa Fluor 594-conjugated goat anti-rabbit IgG (1:200, Cat. No: A11012, Invitrogen) for 2 h at room temperature.

#### RPE/choroid flatmount staining

Mouse eyes were fixed in 2% PFA for 2 h at room temperature. RPE/choroid flatmounts were blocked with 10% goat serum in 5% BSA, permeabilized with 1% triton X-100 for 2 h, followed by incubation with rabbit anti-mouse collagen-1 (1:200, Abcam) and rat anti-mouse CD31(1:100, Cat:MA1-40074, Invitrogen) at 4 °C overnight. After thorough washes, samples were incubated with Alexa Fluor 594-conjugated goat anti-rabbit IgG and Alexa Fluor 488 -conjugated donkey anti-Rat IgG for 2 h.

#### BMDM staining

BMDMs were fixed and permeabilized with pre-cold methanol on ice for 15 min. The cells were incubated with rabbit anti-collagen-1 (1:200, Abcam) and mouse anti-αSMA (1:400, Cat: ab7817, Abcam) at 4 °C overnight, followed by incubation with Alexa Fluor 488 goat anti-ribbit IgG (1:500, Invitrogen) and Alexa Fluor 594 goat anti-mouse IgG (1:500, Cat: A11005, Invitrogen) for 1 h.

Blood and BM cells from young and aged mice with subretinal fibrosis were fixed and permeabilized with pre-cold methanol on ice for 10 min. The samples were blocked and incubated with rat anti-mouse CD45 and rabbit anti-mouse collagen-1 followed by Alexa Fluor 594-conjugated goat anti-rabbit IgG and Alexa Fluor 488-conjugated donkey anti-rat IgG as described above. Cells were then re-suspended in 350 µl stain buffer and transferred onto slides using cytospin ROTOFIX 32A (Hettich, Kirchingen, Germany).

All samples were mounted with 4′,6-Diamidino-2-phenylindole (DAPI)/Anti-fade solution (Cat: S7113, Sigma Aldrich) and imaged using the Zeiss LSM 880 Confocal Microscope (Zeiss, Braunschweig, Germany).

### Image analysis

Three to five images were taken from subretinal fibrosis lesions in cryosections from each eye (*n* = 5 eyes). The percentage of CD45^+^collagen-1^+^cells to total CD45^+^ cells and F4/80^+^collagen-1^+^cells to total F4/80^+^ cells, the number of CD45^+^ collagen-1^+^cells and F4/80^+^ collagen-1^+^cells per mm^2^ lesion area were counted, the number of CD45^+^cells in each lesion and per mm^2^ lesion area were measured. The collagen-1^+^ fibrosis lesion area in the eyes of each group was measured in confocal images of RPE-choroid flatmounts.

Three images were taken from each well of BMDM cultures. The number and percentage of α-SMA^+^collagen-1^+^ cells in BMDMs were manually counted. Twelve to fourteen images were taken from cytospin blood or BM cells from each group. The number and percentage of CD45^+^collagen-1^+^ cells were manually counted. All image analyses were conducted by two researchers in a double-blinded fashion.

### Statistical analysis

GraphPad Prism (Version 8, GraphPad Software, San Diego, CA, USA) was used for statistical analysis. All data were expressed as mean ± SD. The difference between two groups was conducted using the independent Student’s *t* test or paired *t* test (when appropriate). One-way ANOVA was used when comparing multiple groups followed by Tukey’s multiple comparisons for the post-hoc test. Person correlation test was used to evaluate the relationship between the percentage of fibrocyte at the lesion and in the BM or blood, or the relationship between retinal lesion size and BM/blood fibrocytes. *p* < 0.05 was considered statistically significant.

## Results

### Subretinal fibrosis in young and aged mice

**Fig. 1:**
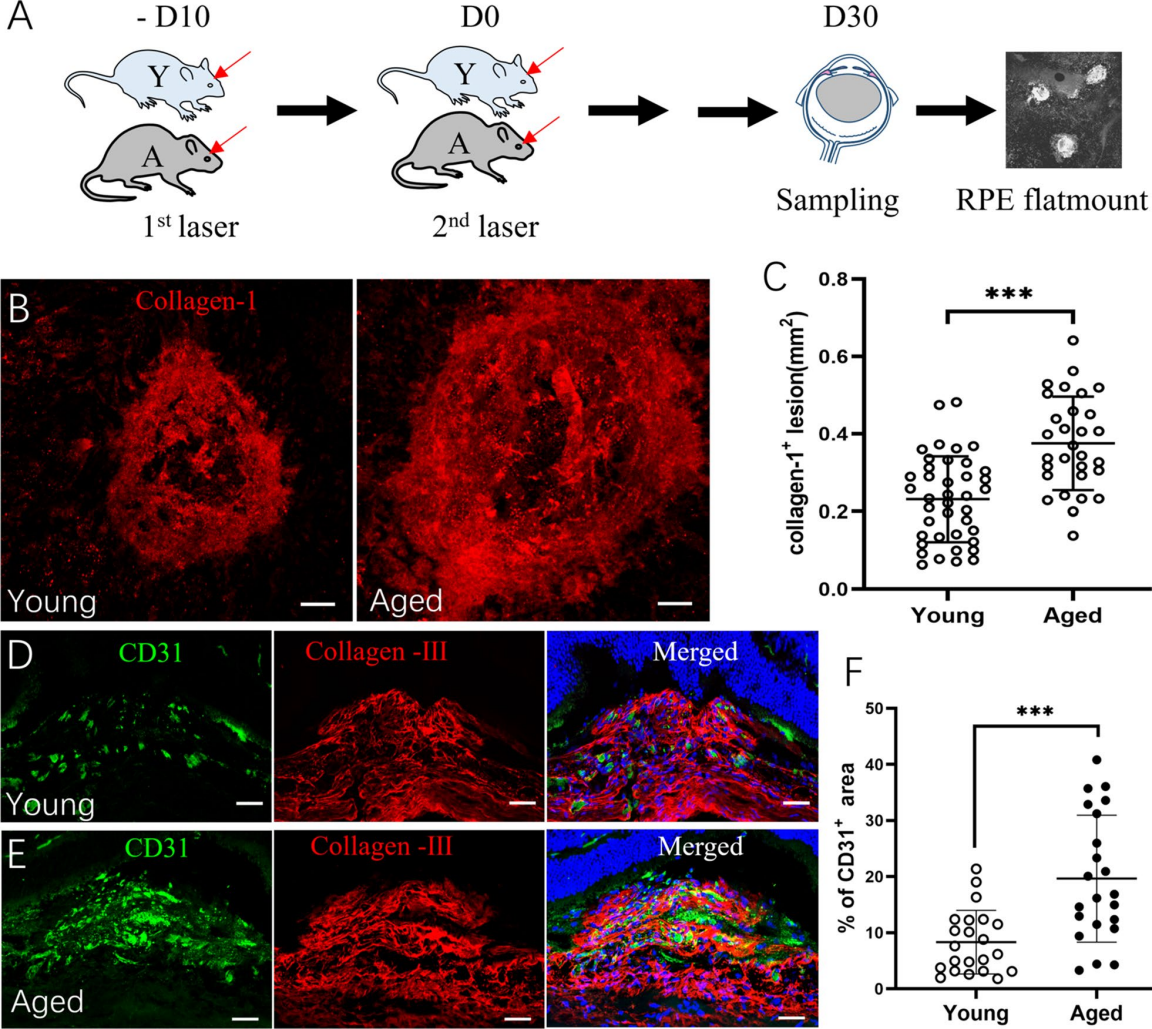
Subretinal fibrosis in young and aged mice. **A** Schematic view of the study design. Mice received two laser burns 10 days apart, eyes were collected 30 days after the second laser for immunohistochemistry. **B** Representative confocal images of RPE/choroid flatmounts stained for collagen-1 from young (2.5-month) and aged (15–16-month) mice. **C** Quantitative analysis of collagen-1^+^ lesion area in young and aged group. **D**, **E** Representative confocal images of retinal cryosections from young (**D**) and aged mice (**E**) with subretinal fibrosis stained for CD31 (green) and collagen-III (red). Cell nuclei were stained with DAPI (blue). **F** Percentage of CD31^+^ blood vessel area in collagen-III^+^ fibrotic lesions of young and aged mice. Mean ± SD, *n* = 30–40 lesions per group from 8 to 10 eyes in **C**, n = 22 lesions per group from 5 to 6 eyes, ***p < 0.001, Student *t* test. Scale bar = 50 µm

Subretinal fibrosis was induced in young (2.5-month) and aged (15–16-month) mice using the two-stage laser protocol [26, 27]. Lesions were examined 30 days after the second laser in RPE/choroid flatmounts (Fig. 1A). Confocal microscopy showed that aged mice had a significantly larger size of the collagen-1^+^ subretinal fibrotic lesion compared with young mice (Fig. 1B, C). The average size of subretinal fibrosis in aged mice (0.376 ± 0.121 mm^2^) was 62% larger than that in young mice (0.231 ± 0.110 mm^2^) (Fig. 1C). Further analysis using anti-CD31 (to identify blood vessels) and anti-collagen III (as an additional marker of fibrosis) immunostaining showed that the fibrotic lesion from aged mice had a significantly larger coverage of CD31^+^ blood vessels compared to that from young mice (Fig. 1D–F).

### Fibrocytes in young and aged mice with or without subretinal fibrosis

**Fig. 2:**
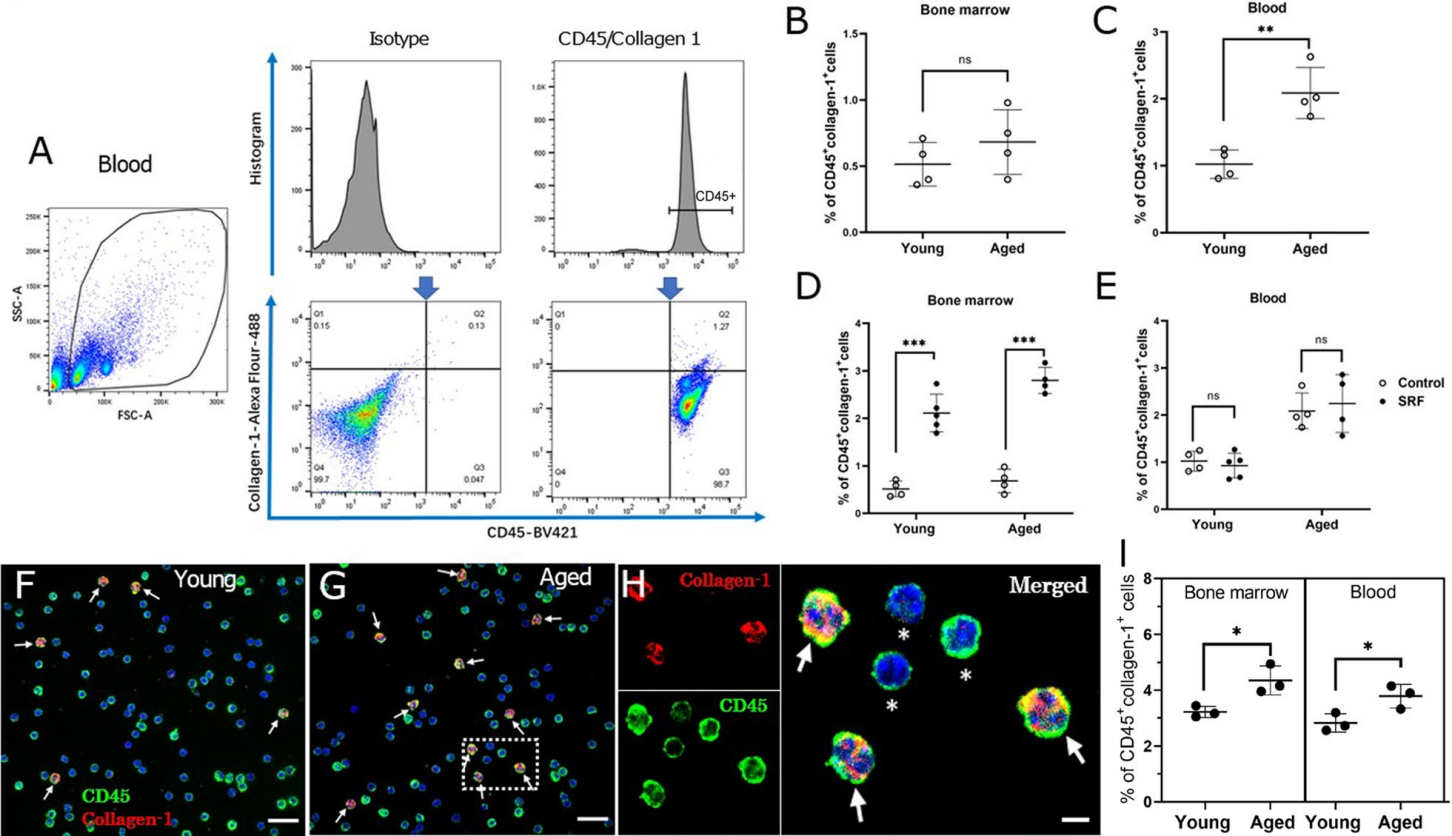
Bone marrow and blood fibrocytes in young (2.5-month) and aged (15–16-month) mice. **A** Gating strategies of blood fibrocytes. The gating strategies were based on relevant isotype controls. CD45^+^Collagen-1^+^ cells were considered circulating fibrocytes. **B**, **C** Percentage of CD45^+^collagen1^+^ fibrocytes in bone marrow (**B**) and blood (**C**) from young and aged mice under normal conditions. **D**, **E** Percentage of CD45^+^collagen1^+^ fibrocytes in bone marrow (**D**) and blood (**E**) from young and aged mice with/without subretinal fibrosis (SRF). Mean ± SD, n = 4–5, **p < 0.01, ***p < 0.001, Student *t* test. **F**, **G** Representative confocal images of blood cells stained for CD45 (green) and collagen-1 (red) from young (**F**) and aged mice (**G**) with subretinal fibrosis. Cell nuclei were stained with 4',6-diamidino-2-phenylindole (DAPI, blue), Scale bar = 20 um. (H) Magnified area of box in (G) showing CD45^+^collagen1^+^ cells (arrows) and CD45^+^collagen1^−^ cells (asterisks). Scale bar = 5 µm. **I** Percentage of CD45^+^collagen1^+^ fibrocytes detected by immunocytochemistry in the bone marrow and blood from young and aged mice with subretinal fibrosis. Mean ± SD, *n* = 3, *p < 0.05, Student *t* test

Fibrocytes in the BM and blood were identified by flow cytometry using CD45 and collagen-1 as markers [22] (Fig. 2A). Under normal conditions, young and aged mice had comparable number of CD45^+^collagen1^+^ fibrocytes in the BM (Fig. 2B), but aged mice had a significantly higher number of fibrocytes in the blood compared to young mice (Fig. 2C).

After induction of subretinal fibrosis, the population of CD45^+^collagen1^+^ cells in the BM, but not blood was significantly increased in both young and aged mice (Fig. 2D, E). Aged subretinal fibrosis mice had a significantly more CD45^+^collagen1^+^ fibrocytes in the BM compared to young subretinal fibrosis mice (2.80 ± 0.27 vs 2.11 ± 0.38,* P* = 0.02) and blood (2.25 ± 0.61 vs 0.93 ± 0.26, *p* < 0.01). This result was further confirmed by immunocytochemistry (Fig. 2F–I).

**Fig. 3:**
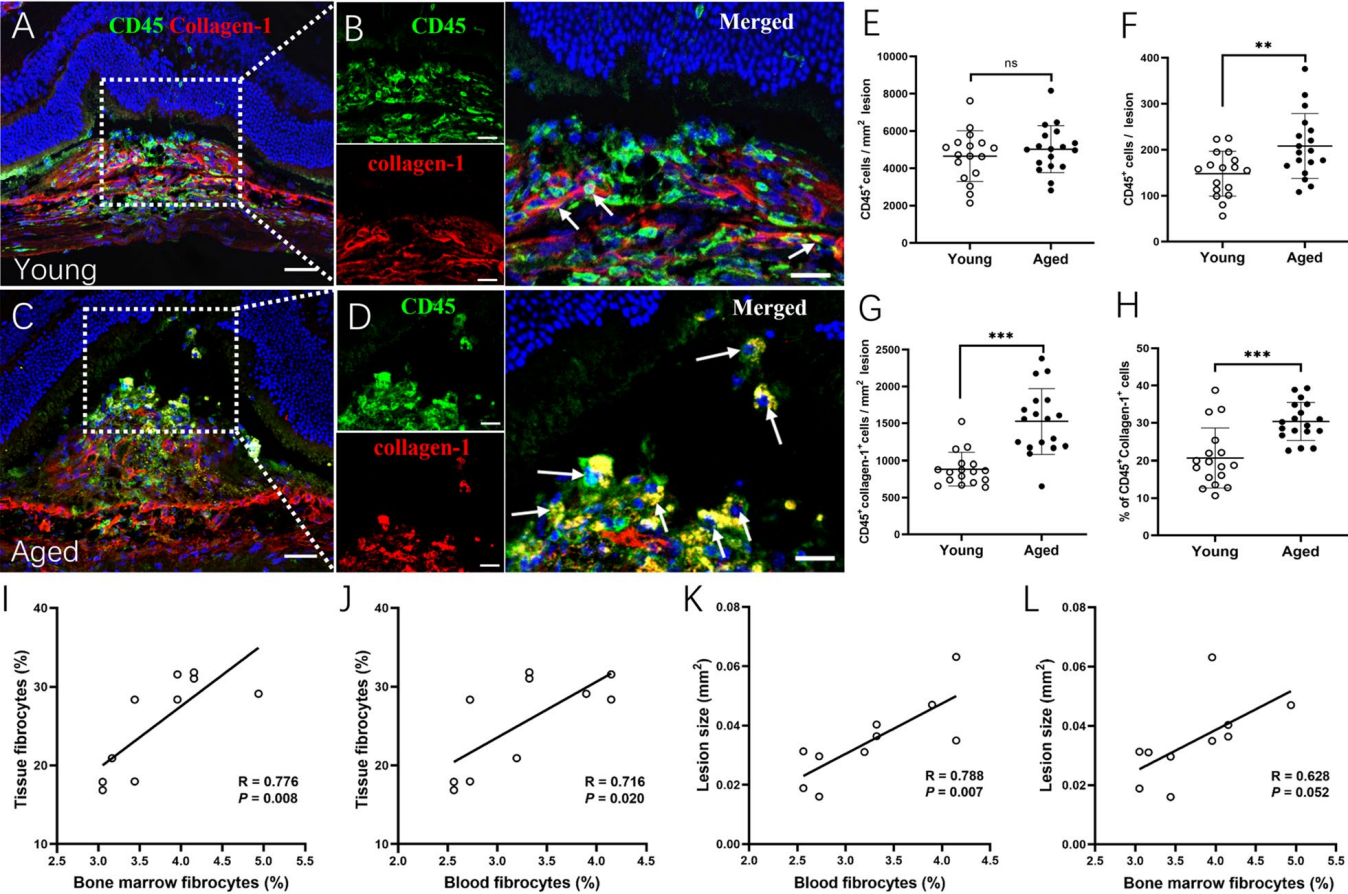
Fibrocytes in the subretinal fibrotic lesion in young and aged mice. **A** Representative confocal image of retinal cryosection from young (2.5-month) mice with subretinal fibrosis stained for nucleus (blue), CD45 (green) and collagen-1 (red). Cell nuclei were stained with 4',6-diamidino-2-phenylindole (DAPI, blue). (**B**) Magnified area of box in **A** showing CD45^+^collagen-1^+^ cells (arrows) in fibrotic lesion. **C** Representative confocal image of retinal cryosection from aged (15–16-month) mice with subretinal fibrosis stained for CD45 (green) and collagen-1 (red). (**D**) Magnified area of box in **C** showing CD45^+^collagen-1^+^ cells (arrows) in fibrotic lesion. **A**, **C** Scale bar = 50 µm; **B**, **D** Scale bar = 20 µm. **E, F** Number of CD45^+^ cells expressed per mm^2^ area of lesion (**E**) and per lesion (**F**) in young and aged mice. **G**, **H** Number of CD45^+^collagen-1^+^ cells in subretinal fibrotic lesions of young and aged mice. **H** Percentage of CD45^+^collagen-1^+^ cells among all CD45^+^ cells in subretinal fibrotic lesions of young and aged mice. Mean ± SD, *n* = 17–18 lesions per group from 5 eyes, **p < 0.01, ***p < 0.001, Student *t* test. **I**, **J** Correlation in the number of CD45^+^collagen-1^+^ cells between retinal fibrotic lesion and bone marrow (**I**) or blood (**J**). **K**, **L** Correlation between subretinal fibrotic lesion size and CD45^+^collagen-1^+^ cells in the bone marrow (**I**) or blood (**J**)

The CD45^+^collagen-1^+^ cells were also detected at the lesion site in young and aged mice (Fig. 3A–D). The number of CD45^+^ cells per mm^2^ lesion was comparable between young and aged mice, but the total number of CD45^+^ cells per lesion was significantly higher in aged mice than that in young mice (Fig. 3E, F). CD45^+^collagen-1^+^ cells constitute ~ 19% of CD45^+^ cells at the lesion in young mice and this number increased to 30% in aged mice (*P* < 0.001, Fig. 3G, H). The number of CD45^+^collagen-1^+^ cells in the fibrotic lesion positively correlated with that in the bone marrow (Fig. 3I) and blood (Fig. 3J). We also found a positive correlation between the fibrotic lesion size and CD45^+^collagen-1^+^ fibrocytes in the blood (Fig. 3K). The correlation between lesion size and bone-marrow CD45^+^collagen-1^+^ cells did not reach statistical significance (Fig. 3L).

### Macrophage-to-myofibroblast transition in BMDMs from young and aged mice

**Fig. 4:**
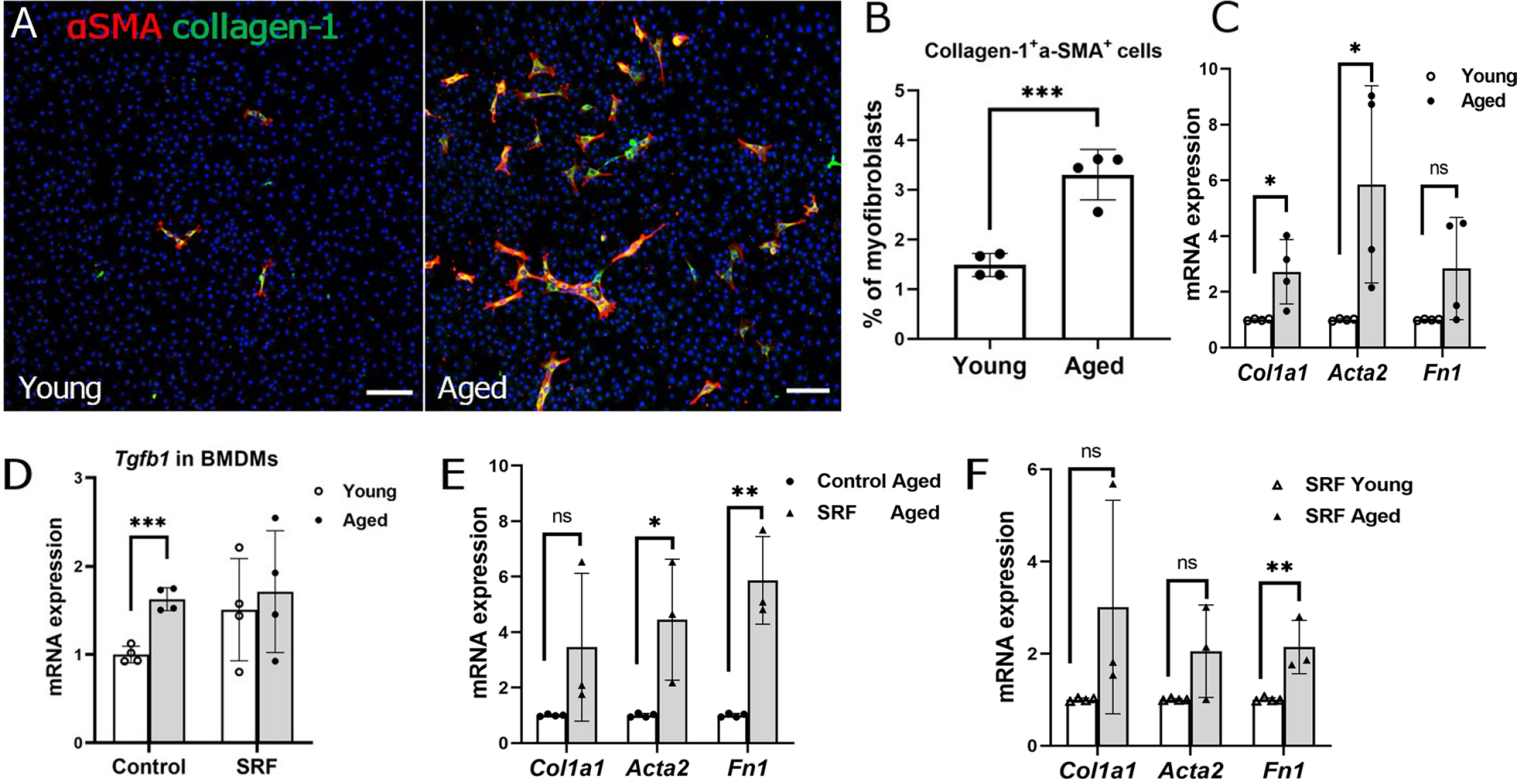
Expression of fibrotic markers in bone-marrow-derived macrophages (BMDMs) from young and aged mice. Primary bone-marrow cells from young and aged mice were isolated and cultured with 20% L929 supernatant for 5–7 days to generate BMDMs for immunocytochemistry or qRT-PCR. **A** Representative confocal images of BMDMs stained for DAPI (blue), α-SMA (red) and collagen-1 (green) from young (2.5-month) and aged (15–16-month) mice under normal conditions. Scale bar = 100um. **B** Dot/bar figure showing the percentages of collagen-1^+^αSMA^+^ cells in young and aged groups. Mean ± SD, n = 4, ***p < 0.001, Student *t* test. **C**–**F** qRT-PCR analysis of the expression of fibrotic marker genes (*Col1a1, Acta2, Fn1*) and *Tgfb1* in BMDMs from normal young and aged mice with or without subretinal fibrosis. **C** Expression of *Col1a1, Acta2,* and *Fn1* genes in the BMDMs from normal young and aged mice. **D** Expression of the *Tgfb1* gene in the BMDMs from normal young and aged mice. **E** Expression of *Col1a1, Acta2,* and *Fn1* genes in the BMDMs from aged mice with and without subretinal fibrosis (SRF). **F** Expression of *Col1a1, Acta2,* and *Fn1* genes in the BMDMs from young and aged mice with subretinal fibrosis. Mean ± SD, *n* = 3–4, *p < 0.05, **p < 0.01,***p < 0.001 Student t test

We previously showed that macrophage-to-myofibroblast transition (MMT) is involved in subretinal fibrosis [29]. Immunocytochemistry showed that the BMDMs from normal aged mice had a higher percentage of collagen-1^+^α-SMA^+^ cells compared with normal young mice (Fig. 4A, B). Quantitative real-time RT-PCR (qRT-PCR) showed that the expression levels of myofibroblast markers collagen-1 (*Col1a1*), α-SMA (*Acta2*) in BMDMs from aged mice were significantly higher than those from young mice, and a trend of increase in fibronectin (*Fn1*) expression (Fig. 4C). Interestingly, BMDM from aged mice also expressed a significantly higher level of *Tgfb1* than that from young mice (Fig. 4D).

After induction of subretinal fibrosis, the expression of *Acta2* and *Fn1* was significantly increased in BMDM from young [27] and aged mice (Fig. 4E), but the expression of *Fn1* in BMDMs from aged subretinal fibrosis mice was significantly higher than that from young subretinal fibrosis mice (Fig. 4F).

**Fig. 5:**
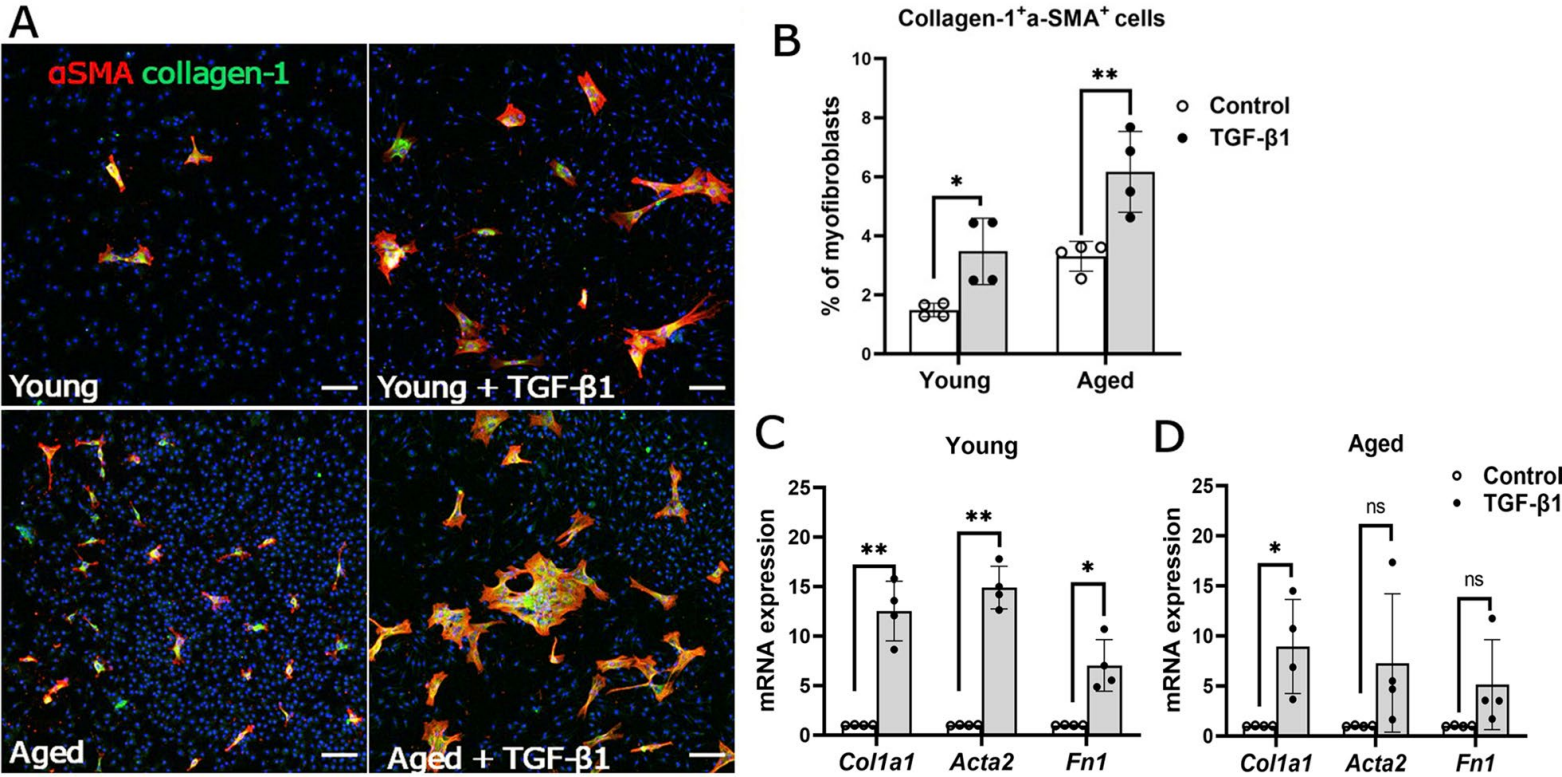
Macrophage-to-myofibroblast transition in BMDMs from normal young (2.5-month) and aged (15–16-month) mice. BMDMs cultured from normal young and aged mice were treated with/without 10 ng/ml of recombinant TGF-β1 for 96 h, and the cells were collected for immunocytochemistry or qRT-PCR. **A** Representative confocal images of control and TGF-β1-treated BMDMs stained for DAPI (blue), α-SMA (red) and collagen-1 (green) from and aged mice. Scale bar = 100 µm. Dot/bar figure showing the percentages of collagen-1^+^ α-SMA^+^ cells in control and TGF-β1-treated BMDMs from young and aged mice. Mean ± SD, *n* = 4, *p < 0.05, **p < 0.01, Student *t* test. **C**, **D** Relative mRNA expression of fibrotic marker genes (*Col1a1, Acta2, Fn1*) in control and TGF-β1-treated BMDMs from young (**C**) and aged mice (**D**). Mean ± SD, *n* = 3–4, *p < 0.05, **p < 0.01, Student *t* test

TGF-β1 treatment markedly increased the percentage of collagen-1^+^α-SMA^+^ cells (Fig. 5A, B) and upregulated the expressions of *Col1a1*, *Acta2* and *Fn1* in both young (Fig. 5C) and aged (Fig. 5D) BMDMs.

Taken together, our results suggest that BMDMs from aged mice had a higher potency to undergo MMT with or without the induction of subretinal fibrosis in vivo or other growth factors, such as TGF-β1 treatment in vitro. In addition, TGF-β1 may promote MMT and BMDMs from aged mice express higher levels of *Tgfb1* compared to BMDMs from young mice.

**Fig. 6:**
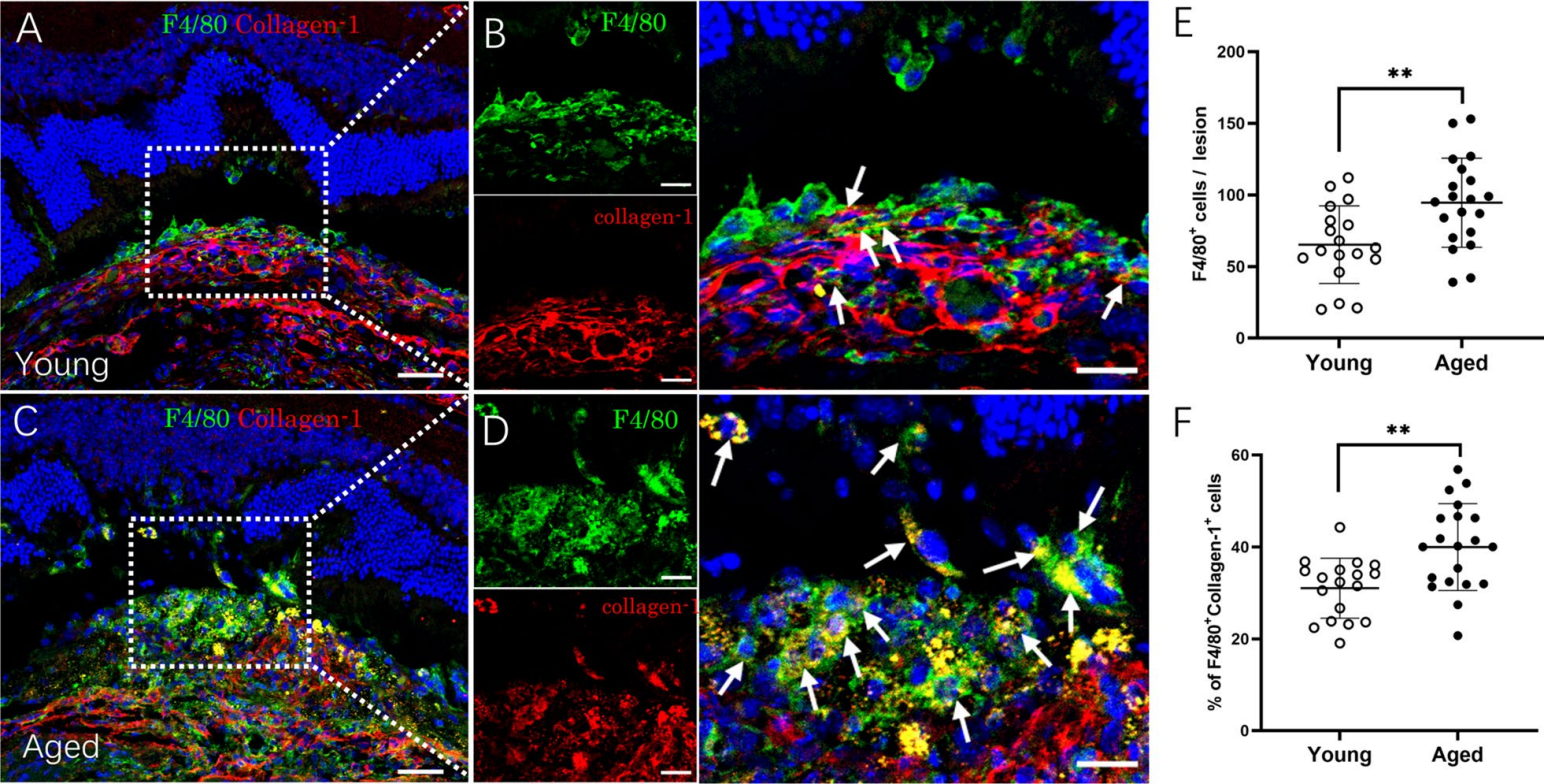
Macrophage to myofibroblast transition (MMT) in the subretinal fibrotic lesion in young and aged mice. **A**–**D** Representative confocal image of retinal cryosections from young (2.5-month, **A**, **B**) and aged (15–16-month, **C**, **D**) mice with subretinal fibrosis stained for F4/80 (green) and collagen-1 (red). Cell nuclei were stained with DAPI (blue). Images in **B** and **D** showing magnified area of boxes in **A** and **C**. Arrows indicating F4/80^+^collagen-1^+^ cells. Scale bar = 50 µm in (**A** and **C**); and 20 µm in **B**, **D**. **E** Number of F4/80^+^ cells in subretinal fibrotic lesion of young and aged mice. **F** Percentage of F4/80^+^collagen-1^+^ cells among all F4/80^+^ cells in subretinal fibrotic lesions of young and aged mice. Mean ± SD, *n* = 18–20 lesions per group from 5 to 6 eyes, **p < 0.01, Student *t* test

We further investigated MMT in fibrotic lesion in young and aged mice. F4/80^+^ macrophages were detected throughout the fibrotic lesion in young and aged mice (Fig. 6A–D). The lesion from aged mice contained a significantly higher number of F4/80^+^ macrophages compared to the lesion from young mice (Fig. 6E). Macrophages undergoing MMT were identified as F4/80^+^collagen-1^+^ (arrows in Fig. 6B, D). The percentage of F4/80^+^collagen-1^+^ cells in the lesion from aged mice was significantly higher than that from young mice (40.0 ± 9.2% vs 31.0 ± 6.4%, *p* < 0.01, Fig. 6E).

### Cytokine/chemokine production in BMDMs from young and aged mice

Multiplex cytokine array assay showed that BMDMs from normal aged mice produced a significantly lower level of CXCL10 than BMDMs from young mice (Additional file 1: Fig. S1A, B). The production of other cytokines including IL-1α, IL-1β, IL-10, VEGF, PDGFα and PDGFβ was comparable between young and aged mice (Additional file 1: Fig. S1A, B). TGF-β1 treatment increased the production of PDGFα, PDGFβ, osteopontin (OPN), and uPAR, and decreased the production of IL-10 and CXCL10 in BMDMs from both young and aged (Table 1) mice. BMDMs from aged mice also produced significantly more GM-CSF, G-CSF, IL-1α, IL-1β, CD105, and EGF following TGF-β1 treatment (Table 1). The production of EGF and OPN in TGF-β1-treated BMDMs from aged mice was significantly higher than that of young mice (Additional file 1: Fig. S1C, D).

**Table 1:** Cytokine/chemokines production control and TGF-β1-treated BMDMs from young and aged mice

Cytokines	Young		Aged	
	Control	TGF-β (+)	Control	TGF-β (+)
GM-CSF	0.94 ± 0.33	1.03 ± 0.07	0.78 ± 0.10	1.00 ± 0.04^*^
G-CSF	1.01 ± 0.45	1.14 ± 0.03	0.76 ± 0.12	1.07 ± 0.17^*^
IL-1α	3.96 ± 1.22	5.80 ± 0.59	3.40 ± 0.54	5.08 ± 0.73^*^
IL-1β	16.44 ± 6.44	19.10 ± 1.91	12.53 ± 2.28	16.87 ± 3.64^*^
IL-10	6.31 ± 1.71	1.29 ± 0.12^*^	4.01 ± 1.07	1.17 ± 0.15^*^
CD105	12.36 ± 4.50	11.83 ± 1.21	9.58 ± 1.35	12.69 ± 1.66^**^
EGF	0.37 ± 0.11	0.42 ± 0.03	0.25 ± 0.06	0.89 ± 0.35^*^
PDGFα	218.34 ± 55.69	856.18 ± 103.05^**^	166.56 ± 19.76	821.05 ± 143.99^**^
PDGFβ	30.62 ± 9.36	123.50 ± 4.51^***^	20.82 ± 3.17	91.04 ± 22.62^**^
CCL2	160.12 ± 56.04	65.62 ± 8.95^*^	143.38 ± 71.02	88.03 ± 27.11
VEGF	402.54 ± 147.91	263.22 ± 78.01	324.79 ± 42.49	241.30 ± 106.44
CXCL10	577.83 ± 44.05	33.56 ± 8.63^***^	321.68 ± 119.46	31.57 ± 4.75^*^
OPN	4159.76 ± 3108.26	7469.01 ± 1454.25^*^	2477.5 ± 1083.4	9403.29 ± 1462.13^***^
Ang-2	136.67 ± 71.45	108.87 ± 15.26	112.36 ± 38.47	105.50 ± 12.34
uPAR	44.94 ± 15.75	330.99 ± 41.08^**^	38.70 ± 14.06	398.47 ± 165.26^*^

Cytokine/growth factor values were normalized to total protein levels of the samples and expressed as pg/total protein. Mean ± SD, n = 4, *p < 0.05; **p < 0.01; ***p < 0.001 compared to control non-TGF-β1-treated cells of the same age group, paired *t* test

After induction of subretinal fibrosis, BMDMs from aged mice showed a trend of increment in the production of CCL2, VEGF, OPN, and Angiopoietin-2 (Ang-2) compared with BMDMs from young mice (Additional file 1: Fig. S1E, F). The production of Ang-2 in TGF-β1-treated BMDMs from aged subretinal mice was notably higher than that from young subretinal mice (Additional file 1: Fig. S1G, H). We also found that induction of subretinal fibrosis significantly increased the production of uPAR and decreased CXCL10 in BMDMs from young and aged mice, and enhanced the production of VEGF and Ang-2 in BMDMs from aged mice (Table 2).

**Table 2:** Cytokine/chemokines production of BMDMs from young and aged mice with/without subretinal fibrosis

Cytokines	Groups	Control	Subretinal fibrosis	*p* value
CXCL10	Young	577.83 ± 44.05	135.99 ± 35.32	0.000
	Aged	321.68 ± 119.46	154.56 ± 36.34	0.044
uPAR	Young	44.94 ± 15.75	313.91 ± 80.79	0.001
	Aged	38.70 ± 14.06	330.79 ± 72.55	0.001
VEGF	Young	402.54 ± 147.91	377.01 ± 103.45	0.797
	Aged	324.79 ± 42.49	732.84 ± 188.64	0.029
Ang-2	Young	136.67 ± 71.45	170.83 ± 56.14	0.505
	Aged	112.36 ± 38.47	567.41 ± 96.99	0.045

Cytokine/growth factor values were normalized to total protein levels of the samples and expressed as pg/total protein. Mean ± SD, *n* = 4, Student *t* test

Our results suggest that old age drives BMDMs towards a pro-angiogenic and pro-fibrotic phenotype, particularly, under disease conditions.

### The effect of aged bone-marrow-derived cells in the development of subretinal fibrosis in young mice

**Fig. 7:**
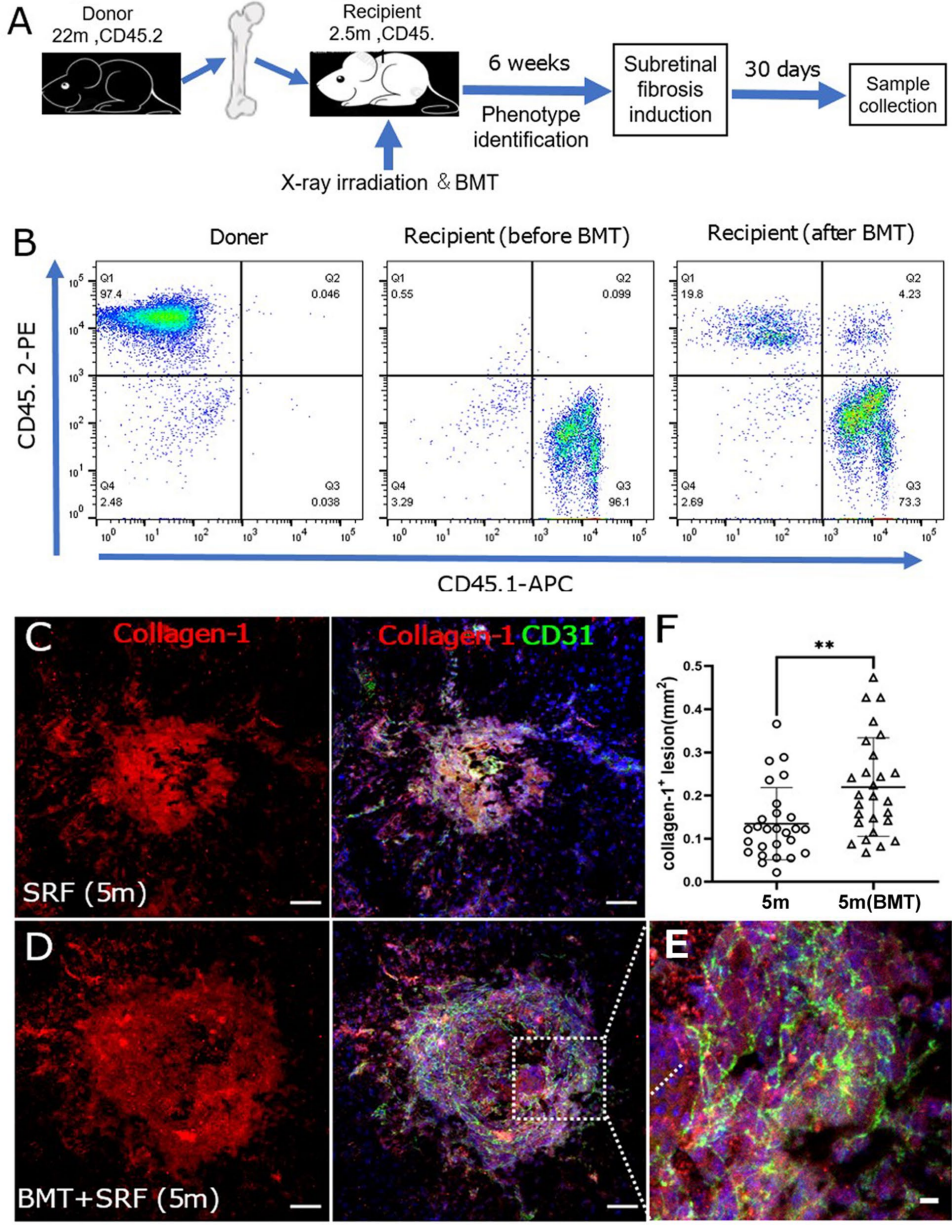
Effect of aged bone-marrow cells in subretinal fibrosis in young mice. **A** Schematic view of the study design. Bone-marrow cells from 22-month Ly5.2 donor mice were transplanted into the young (2.5-month) X-ray irradiated recipient mice via tail vein injection (Aged Ly5.2(CD45.2) → Young Ly5.1 (CD45.1). 6 weeks later, flow cytometry was carried out to assess the immune system reconstitution in the blood of recipient mice. The mice were subjected to subretinal fibrosis induction. Eyes were collected for immunohistochemistry 4 weeks after the second laser. **B** Representative flow cytometry dot plot showing CD45.1 and CD45.2 expression in the aged donor, and young recipient mice before and 6 weeks after BMT. **C**–**D** Representative confocal images of RPE/choroid flatmounts stained for collagen-1 (red) and CD31 (green) from 4-month-old non-BMT young controls (**C**) and BMT young mice (**D**). Cell nuclei were stained with DAPI (blue). Scale bar = 50 um. (E) Magnified area of box in **D** showing CD31^+^ blood vessels in collagen-1^+^ fibrotic lesions. **F** Quantitative analysis of collagen-1^+^ lesion area in 5-month-old non-BMT controls and 5-month-old BMT mice. Mean ± SD, *n* = 27 lesions per group from 7 to 8 eyes, **p < 0.01, Student t test

To further understand the role of aged BM-derived cells in subretinal fibrosis, we transplanted BM cells from 22-month aged mice (CD45.2) into 2.5-month young mice (CD45.1) or from 2.5-month young mice (CD45.1) into 22-month aged mice (CD45.2) [30, 31]. Subretinal fibrosis was induced in recipient mice 6 weeks after BM transplantation (BMT). Eyes were collected 30 days after the second laser treatment (Fig. 7A). Flow cytometry showed that four young recipient mice (CD45.1) had between 5 ~ 68% (average 22.35%) of CD45.2^+^ cells in the blood (Fig. 7B), suggesting a partial reconstitution of the immune system by BM cells from aged mice. The remaining young mice (*n* = 4) had < 2% of CD45.2^+^ cells. All aged recipient mice (*n* = 5, CD45.2) had < 2% of CD45.1^+^ cells (Additional file 1: Fig. S2). The results suggest unsuccessful BMT in the four young and five aged recipient mice. The failure is probably due to insufficient X-ray irradiation as we did not observe a significant drop in body weight and the immune system remained fully functional in these mice. Nevertheless, we proceeded with subretinal fibrosis induction in these mice.

When comparing the size of subretinal fibrosis between partially constituted BMT recipient young mice (received BM cells from aged CD45.2 mice) with age-matched controls, a significantly larger collagen-1^+^ fibrotic lesion was observed in partial BMT mice (Fig. 7C–F). Dual staining of CD31 and collagen-1 showed that the lesion contains a network of CD31^+^ blood vessels (Fig. 7E). Our results suggest that even with partial BM re-constitution, aged BM cells significantly promoted subretinal fibrosis in young recipient mice.

We also examined the subretinal collagen-1^+^ lesions in recipient young (CD45.1) and aged (CD45.2) mice with failed BMT (BMT-f) and compared the lesion size with age-matched controls (i.e., without X-ray irradiation). The fibrotic lesions in normal 25-month-old mice (0.36 ± 0.13 mm^2^) were nearly three times bigger than those in 5-month-old mice (0.13 ± 0.08 mm^2^). Within the same age group, the lesion sizes were comparable between non-irradiated and X-ray irradiated (BMT-f) mice (Additional file 1: Fig. S2B–D).

Since all recipient mice received the same amount of X-ray irradiation, our results suggest that irradiation per se did not affect subretinal fibrosis and increased subretinal fibrosis in young mice received BM from old mice (Fig. 7C–F) is due to intrinsic profibrotic potential of the old BM.

## Discussion

**Fig. 8:**
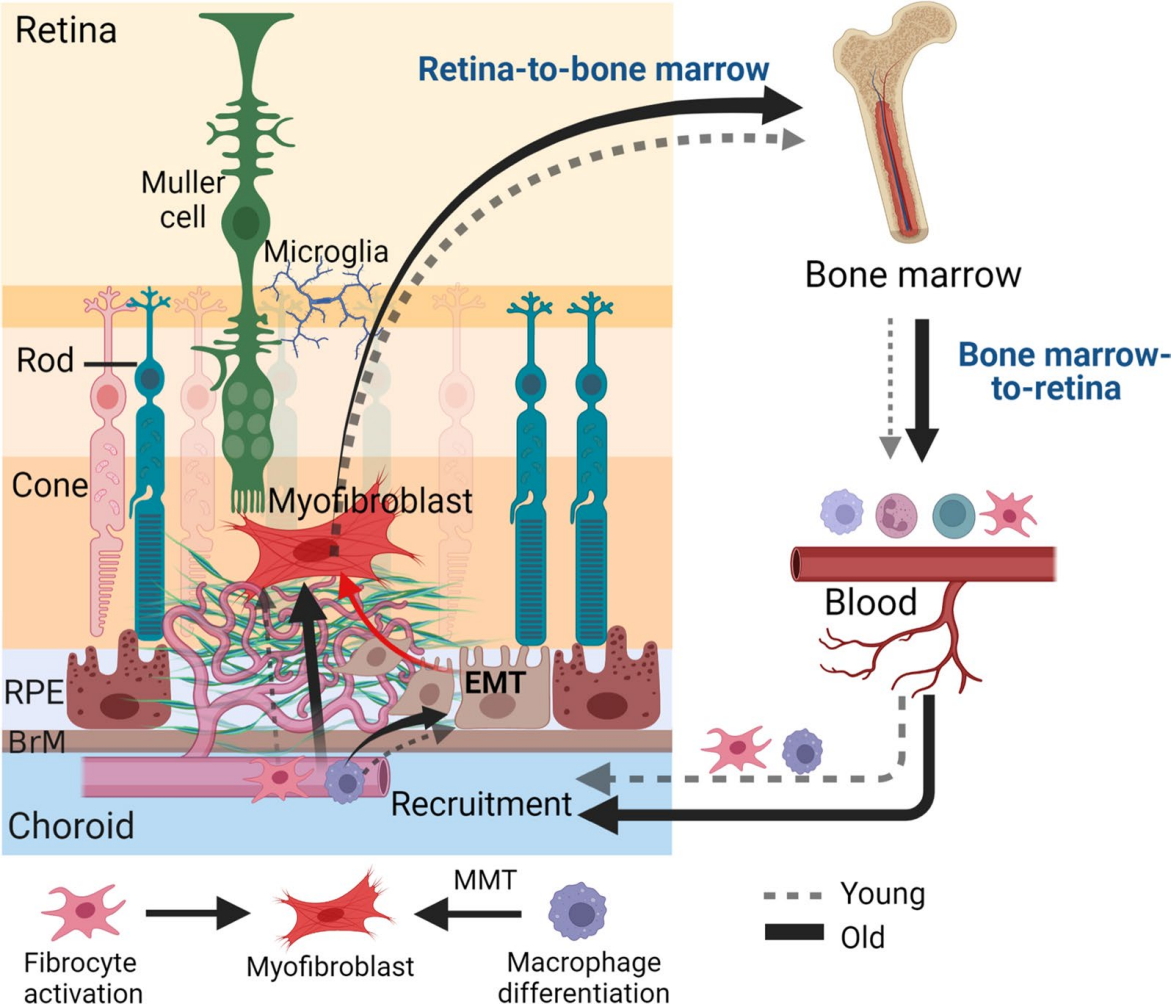
Retina–bone-marrow–blood–retina pathway of fibrocytes and macrophage recruitment in the development of subretinal fibrosis. Retinal injury is detected by the bone marrow, which then produces fibrocytes and pro-fibrotic macrophages and releases them to the blood circulation. Once recruited into the damaged site, fibrocytes can differentiate into myofibroblasts. Macrophages can release profibrotic factors that promote epithelial-to-mesenchymal transition (EMT) in RPE cells. Macrophages can also participate in subretinal fibrosis through macrophage-to-myofibroblast transition (MMT). This process is augmented during ageing. (Image was created in BioRender.com)

In this study, we show that ageing promotes CNV-mediated subretinal fibrosis. Mechanistically, we found that when retinal injury occurs, there is a retina–BM–blood–retina pathway of fibrocyte and macrophage recruitment and this process is accelerated in aged mice (Fig. 8). Aged mice had a higher number of circulating fibrocytes. Macrophages from aged mice had a higher potency of transdifferentiating into myofibroblasts compared with the cells from young mice. Our findings revealed a previously unrecognized role of BM-derived cells in age-related organ fibrosis.

Myofibroblasts are the key drivers of fibrosis, although their origins vary in different organs. In the case of subretinal fibrosis, it has been suggested that choroidal fibroblasts, pericytes and bone-marrow-derived cells may constitute possible origins [32–34]. Alternatively, RPE cells and vascular endothelial cells may be differentiated into cells of mesenchymal phenotype through the epithelial–mesenchymal transition (EMT) and endothelial–mesenchymal transition (EndoMT), respectively [32, 35]. It is well-known that ageing impairs tissue repair and wound healing and increases the risk of fibrosis, although the underlying mechanism remains poorly defined.

Macrophages are critically involved in all stages of wound healing (inflammation, proliferation and remodeling) and can act as a potential source of myofibroblasts through MMT. Ageing reduces many functions of macrophages including TLR expression, phagocytosis, bactericidal activity, cytokine and chemokine secretion [36], which may be responsible for age-related decline in wound healing. We found that macrophages from aged mice express higher levels of *Tgfb1* (Fig. 4), produce more Ang-2, EGF and OPN, but less CXCL10, an anti-fibrotic chemokine [37]. Furthermore, we found that macrophages from aged mice constitutively express higher levels of myofibroblast marker (*Col1a1, Acta2* and *Fn*) and have a higher potency of MMT. Retinal laser injury enhanced the potency of MMT in BMDMs, suggesting the existence of a retina-to-bone-marrow feedback pathway (Fig. 8). The level of MMT in aged mice was significantly higher than that in young mice. Our results suggest that ageing drives macrophages to a pro-fibrotic phenotype and enhances MMT during retinal injury.

We found that fibrocytes are another potential source of myofibroblasts in subretinal fibrosis. Normal aged mice contained significantly more circulating fibrocytes compared to young mice. After induction of subretinal fibrosis, aged mice had significantly higher levels of CD45^+^Collagen-1^+^ fibrocytes in the BM, blood (Fig. 2) and fibrotic lesion (Fig. 3). The number of fibrocytes in the BM and blood positively correlated with that in the lesion and the cells also positively correlated with the lesion size (Fig. 3). Our results suggest that the retina–BM–blood–retina pathway of fibrocyte recruitment is accelerated in aged mice (Fig. 8). Due to the low number of circulating fibrocytes, we were unable to conduct functional analysis in these cells. It is, therefore, unclear how old age may affect fibrocyte functions.

Both circulating monocytes/macrophages and fibrocytes originate from the BM. When we transplanted the BM from aged mice to young mice, it significantly enhanced subretinal fibrosis. Our results suggest that aged BM and their derivatives have a higher pro-fibrotic potential, which may contribute to aggregated subretinal fibrosis secondary to CNV in aged mice. Since macular fibrosis in nAMD is a disease that affects the elderly, it will be important to know if BM-derived fibrocytes and macrophages from aged people, in particular nAMD patients, are also more pro-fibrotic, compared to the cells from young people to develop strategies for therapy.

The study has a number of limitations. First, in vitro analysis was only carried out in macrophages but not fibrocytes due to technical challenges. Further studies using single cell RNA sequencing in young and aged blood cells or circulating fibrocytes from different ages of human donors will help to understand the impact of age on fibrocyte function. Second, we did not distinguish the contribution of BMDM (via MMT) from circulating fibrocytes to subretinal fibrosis. Therefore, we do not know each of their contributions to subretinal fibrosis during ageing. Using macrophage or fibrocyte specific knockout mice or cell depletion studies will help to address the question. In addition, apart from the systemic factors investigated in this study (i.e., BM-derived macrophages and fibrocytes), the tissue microenvironment may also play a role in the age-related worsening of subretinal fibrosis. We and others have shown that a low-grade chronic inflammation (para-inflammation) exists in the ageing choroid [38] and retina [39, 40]. RPE cells in the aged mice are multinucleated and more fragile to additional insults and they have impaired wound healing ability [41, 42]. Immune regulation in the ageing retina is also altered and is at a greater risk of being dysregulated [43]. When an injury occurs in the ageing retina, wound healing could be accompanied by severe and dysregulated inflammation [44], which may contribute to aggregated fibrosis.

## Conclusions

Our study suggests that ageing promotes CNV-induced subretinal fibrosis due to increased circulating fibrocytes and the pro-fibrotic potential of BM-derived macrophages. Our study also suggests the existence of a retina–BM–blood–retina signaling pathway of fibrocyte/macrophage recruitment (Fig. 8). Retinal damages are readily detected by the BM. In response to retinal damage, fibrocytes are rapidly mobilized from the BM and recruited to the retina (Fig. 8). In the meantime, BM-derived cells including macrophages are programmed to a wound healing and repair phenotype and express higher levels of mesenchymal markers (*Col1a1, Acta2, and Fn*). This response is more pronounced in ageing conditions (Fig. 8). Further understanding the molecular mechanism of the retina-to-BM signaling pathway and how this is affected by ageing may help to develop better strategies to promote retinal repair and prevent fibrosis, particularly in people with nAMD.

## Supplementary Information

**Additional file 1: Figure S1:** Cytokines/chemokines production in BMDMs from young and aged mice. BMDMs from young (3-month) and aged (16-month) mice were treated with/without mouse recombinant TGF-β1 (10ng/ml) for 96 h. Supernatants were collected and used for Luminex multiplex cytokine assay. All value were normalised by the total protein levels of the sample. (A, B) Production of cytokines and growth factors in the supernatants of BMDMs from normal young and aged mice without TGF-β1 treatment. (C, D) Production of cytokines and growth factors in the supernatants of BMDMs from normal young and aged mice after TGF-β1 treatment. (E, F) Production of cytokines and growth factors in the supernatants of BMDMs from young and aged mice with subretinal fibrosis under normal culture conditions. (G, H) Production of cytokines and growth factors in the supernatants of BMDMs from young and aged mice with subretinal fibrosis after TGF-β1-treated. Mean ± SD, n=4, *p < 0.05 , Student t test. **Figure S2. **Effects of X-ray irradiation on subretinal fibrosis in young and aged mice. (A) Representative flow cytometry data showing CD45.1 and CD45.2 expression in control and unsuccessful bone-marrow transplanted (BMT-f) young (5-month) and aged (25-month) mice. Young BMT mice (CD45.1) received aged BM (CD45.2). Aged BMT mice (CD45.2) received young BM (CD45.1). (B) Representative confocal images of RPE/choroid flatmounts stained for collagen-1(red) from young and aged mice with or without BMT (X-ray irradiated, but no immune system reconstitution by transplanted BM cells). (C, D) Quantitative analysis of subretinal collagen-1^+^ lesion area in young (C) and aged (D) mice with or without X-ray irradiation. Mean ± SD, n = 24~27 lesions per group from 6 to 7 eyes (C), or n = 11~20 lesions per group from 3 to 5 eyes (D), Student t test. **Table S1**. Primer sequences of mouse genes for quantitative RT-PCR.

## Data Availability

The authors confirm that the data supporting the findings of this study are available within the article and supplementary materials.

## Abbreviations

Ang2: Angiopoietin-2
AMD: Age-related macular degeneration
nAMD: Neovascular age-related macular degeneration
ANOVA: Analysis of variance
BM: Bone marrow
BMT: Bone-marrow transplantation
BMDMs: Bone-marrow-derived macrophages
CNV: Choroidal neovascularization
CXCL10: C–X–C motif chemokine ligand 10
DAPI: 4′,6-Diamidino-2-phenylindole
DMEM: *Dulbecco's Modified Eagle medium*
FN: Fibronectin
MMT: Macrophage-to-myofibroblast transition
OPN: Osteopontin
PDGFα: Platelet-derived growth factor-alpha
PDGFβ: Platelet-derived growth factor-beta
qRT-PCR: Quantitative real-time polymerase chain reaction
RPE: Retinal pigment epithelial cells
uPAR: urokinase plasminogen activator surface receptor
VEGF: Vascular endothelial growth factor
αSMA: α-Smooth muscle actin
TGF-β: Transforming growth factor beta
