# Supplementary material for: Old age promotes retinal fibrosis in choroidal neovascularization through circulating fibrocytes and profibrotic macrophages
Source: J Neuroinflammation. 2023 Feb 23;20:45. doi: 10.1186/s12974-023-02731-y (PMC9947907; doi:10.1186/s12974-023-02731-y)
Supplement: Supplementary file 1 — Additional file 1: Figure S1: Cytokines/chemokines production in BMDMs from young and aged mice. BMDMs from young (3-month) and aged (16-month) mice were treated with/without mouse recombinant TGF-β1 (10ng/ml) for 96 h. Supernatants were collected and used for Luminex multiplex cytokine assay. All value were normalised by the total protein levels of the sample. (A, B) Production of cytokines and growth factors in the supernatants of BMDMs from normal young and aged mice without TGF-β1 treatment. (C, D) Production of cytokines and growth factors in the supernatants of BMDMs from normal young and aged mice after TGF-β1 treatment. (E, F) Production of cytokines and growth factors in the supernatants of BMDMs from young and aged mice with subretinal fibrosis under normal culture conditions. (G, H) Production of cytokines and growth factors in the supernatants of BMDMs from young and aged mice with subretinal fibrosis after TGF-β1-treated. Mean ± SD, n=4, *p < 0.05 , Student t test. Figure S2. Effects of X-ray irradiation on subretinal fibrosis in young and aged mice. (A) Representative flow cytometry data showing CD45.1 and CD45.2 expression in control and unsuccessful bone-marrow transplanted (BMT-f) young (5-month) and aged (25-month) mice. Young BMT mice (CD45.1) received aged BM (CD45.2). Aged BMT mice (CD45.2) received young BM (CD45.1). (B) Representative confocal images of RPE/choroid flatmounts stained for collagen-1(red) from young and aged mice with or without BMT (X-ray irradiated, but no immune system reconstitution by transplanted BM cells). (C, D) Quantitative analysis of subretinal collagen-1+ lesion area in young (C) and aged (D) mice with or without X-ray irradiation. Mean ± SD, n = 24~27 lesions per group from 6 to 7 eyes (C), or n = 11~20 lesions per group from 3 to 5 eyes (D), Student t test. Table S1. Primer sequences of mouse genes for quantitative RT-PCR. [file 12974_2023_2731_MOESM1_ESM.docx]

**Additional file 1**


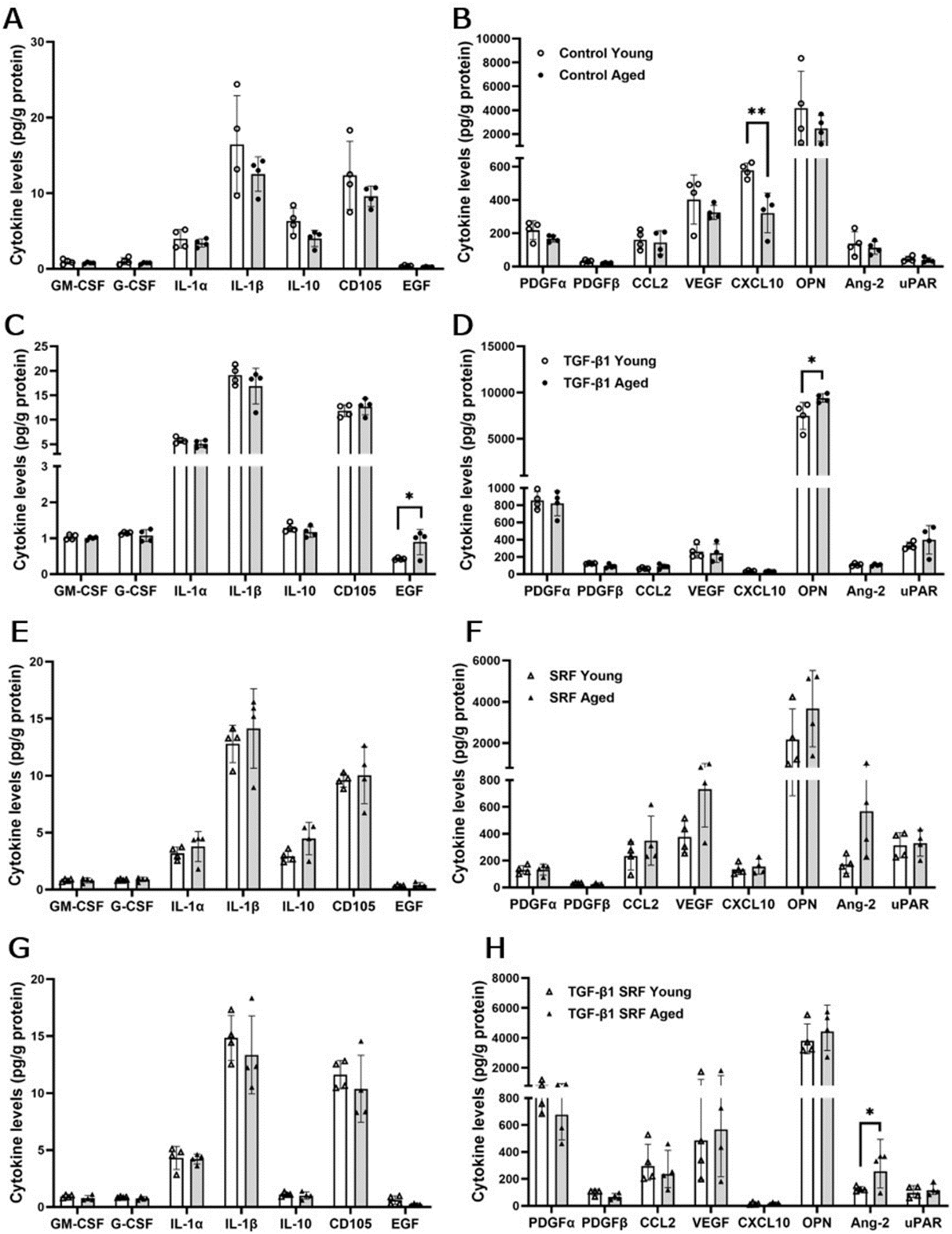


**Figure S1:** Cytokines/chemokines production in BMDMs from young and aged mice. BMDMs from young (3-month) and aged (16-month) mice were treated with/without mouse recombinant TGF-β1 (10ng/ml) for 96 h. Supernatants were collected and used for Luminex multiplex cytokine assay. All value were normalised by the total protein levels of the sample. (A-B) The production of cytokines and growth factors in the supernatants of BMDMs from normal young and aged mice without TGF-β1 treatment. (C - D) The production of cytokines and growth factors in the supernatants of BMDMs from normal young and aged mice after TGF-β1 treatment. (E - F) The production of cytokines and growth factors in the supernatants of BMDMs from young and aged mice with subretinal fibrosis under normal culture conditions. (G - H) The production of cytokines and growth factors in the supernatants of BMDMs from young and aged mice with subretinal fibrosis after TGF-β1 treated. Mean ± SD, n=4, **p* < 0.05 , Student t test.


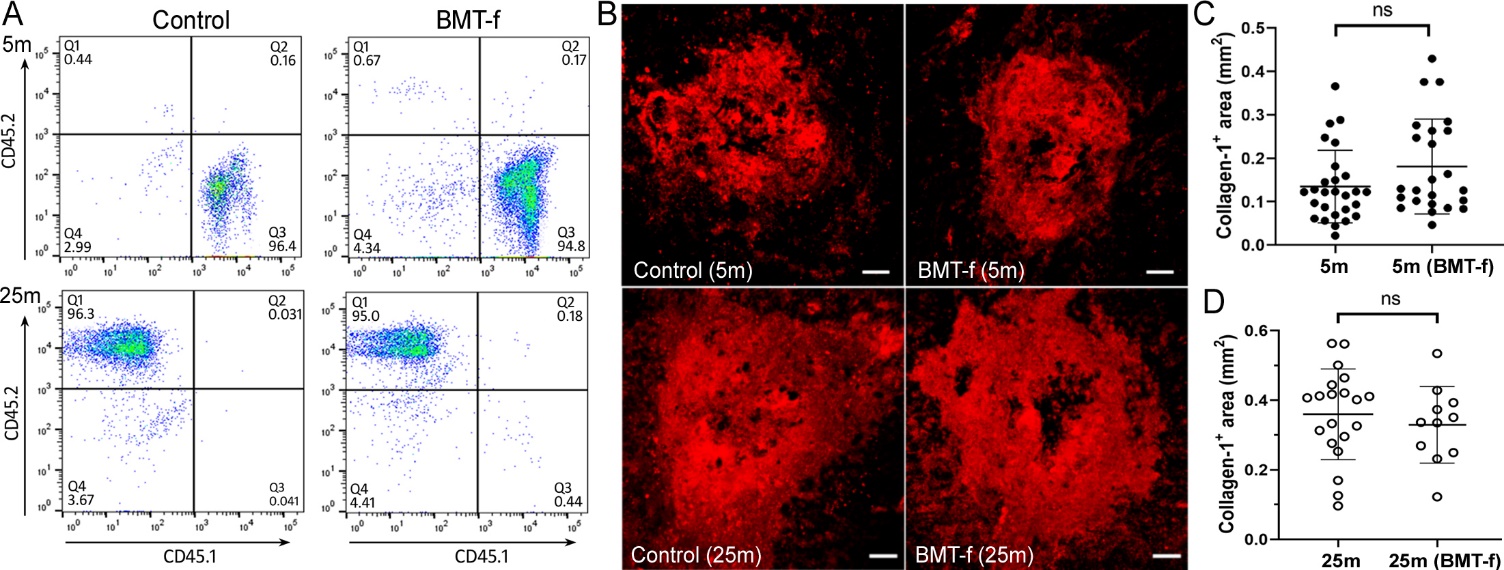


**Figure S2. Effects of X-ray irradiation on subretinal fibrosis in young and aged mice.** (A) Representative flow cytometry data showing CD45.1 and CD45.2 expression in control and unsuccessful bone marrow transplanted (BMT-f) young (5-month) and aged (25-month) mice. Young BMT mice (CD45.1) received aged BM (CD45.2). Aged BMT mice (CD45.2) received young BM (CD45.1). (B) Representative confocal images of RPE/choroid flatmounts stained for collagen-1(red) from young and aged mice with or without BMT (x-ray irradiated, but no immune system reconstitution by transplanted BM cells). (C - D) Quantitative analysis of subretinal collagen-1^+^ lesion area in young (C) and aged (D) mice with or without x-ray irradiation. Mean ± SD, n = 24~27 lesions per group from 6-7 eyes (C), or n = 11~20 lesions per group from 3-5 eyes (D), Student t test.

**Table S1. Primer sequences of mouse genes for quantitative RT-PCR.**

| **Gene** | **Forward sequence 5'-3'** | **Reverse sequence 5'-3'** |
| --- | --- | --- |
| *Col1a1* | CTGGCGGTTCAGGTCCAAT | TTCCAGGCAATCCACGAGC |
| *Fn1* | GCCGTTAGATGTGCAAGCTG | TGCTGAAGCTGAGAACTAGGC |
| *Acta2* | GGACGTACAACTGGTATTGTGC | TCGGCAGTAGTCACGAAGGA |
| *Tgfb1* | CATCCATGACATGAACCGGC | GAAGTTGGCATGGTAGCCCT |
| *GAPDH* | CTCAGGAGAGTGTTTCCTCGTC | ATGGGCTTCCCGTTGATGAC |
